# Prevalence, trends, and inequality in noncommunicable diseases in Bangladesh: Evidence from Bangladesh Demographic and Health Surveys 2011 and 2017–2018

**DOI:** 10.1002/puh2.148

**Published:** 2024-01-15

**Authors:** Masum Ali, Md. Ruhul Amin, Johan Jarl, Sanjib Saha

**Affiliations:** ^1^ Poverty, Gender, and Inclusion International Food Policy Research Institute (IFPRI) Dhaka Bangladesh; ^2^ Institute of Nutrition and Food Science (INFS) University of Dhaka Dhaka Bangladesh; ^3^ Health Economics Unit Department of Clinical Science (Malmö) Lund University Lund Sweden

**Keywords:** diabetes Bangladesh, hypertension, noncommunicable diseases, overweight

## Abstract

We investigated the change of the prevalence of noncommunicable diseases (NCDs) in Bangladesh from 2011 to 2018 across different socioeconomic groups as well as the factors associated with the changes in prevalence. We used the two waves of the Bangladesh Demographic and Health Surveys conducted in 2011 and 2017–2018. Modified Poisson regression model was used to estimate the prevalence rate and ratio of NCDs and to test the association with different demographic and socioeconomic variables. The study found an upward trend of NCDs from 2011 to 2017 in which overweight and obesity, hypertension, and diabetes increased by 1.8, 1.5, and 1.1 times, respectively. In 2011, people from the richest households had 5.6 higher odds of being overweight compared to the poorest, which was reduced to 3.0 in 2017. However, the increment for overweight and hypertension was the highest among the poor and manual workers from 2011 to 2017. The age‐adjusted prevalence ratio of overweight increased 4.4 times for the poorest, compared to 1.7 times for the richest. For manual workers, overweight increased 3.8 times, whereas hypertension increased by 2.4 times. The pooled analysis revealed that participants from the richest households have the highest risk of NCDs, with 3.3 times for overweight, 2.3 times for diabetes, and 1.3 times for hypertension, compared to the poorest. However, the prevalence of NCDs is rising quickly among the low socioeconomic groups in Bangladesh, narrowing the gap with higher socioeconomic groups. Our findings call for immediate policy interventions and targeted programs to curb NCD escalation in Bangladesh.

## Graphical abstract

Background: We investigated the change of the prevalence of noncommunicable diseases (NCDs) in Bangladesh from 2011 to 2018 across different socioeconomic groups as well as the factors associated with the changes in prevalence.Data source: We used the two waves of the Bangladesh Demographic and Health Surveys conducted in 2011 and 2017–2018.Method: Modified Poisson Regression model was used to estimate the prevalence rate and ratio of NCDs and to test the association with different demographic and socioeconomic variables.Result: Overweight OR = 3.3 95% CI (2.8–3.9); diabetes OR = 2.3 95% CI (1.9–2.7); hypertension = 1.3 95% CI (1.2–1.4).Conclusion: The prevalence of NCDs is rising quickly among the low socioeconomic groups in Bangladesh, narrowing the gap with higher socioeconomic groups. Our findings call for immediate policy interventions and targeted programs to curb NCD escalation in Bangladesh.

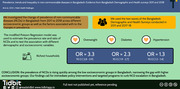

## INTRODUCTION

Noncommunicable diseases (NCDs) pose a great threat to global public health in terms of morbidity, mortality, and healthcare cost. NCD‐related mortality is high in low‐ and middle‐income countries (LMICs), whereas NCDs are prominent among individuals in lower socioeconomic status population groups in high‐income countries, exacerbating both global and national health inequalities [1]. NCDs are responsible for 41 million deaths each year, which is equivalent to 71% of all deaths in the world. Thirty seven percent of NCD deaths occurred among people aged 30–69 years and of those deaths, 85% occur in LMICs [2]. It is estimated that the global burden of NCDs will increase by 17% over the next decade, with the greatest share occurring in LMICs. The effect of globalization and urbanization on LMICs has fast‐tracked the increasing burden of NCDs. Yet, governments in LMICs are not keeping pace with escalating requirements for policies, laws, regulations, facilities, and infrastructure to combat NCDs.

In Bangladesh, NCDs accounted for 70% of all deaths in 2019 [3]. The country is experiencing a dual disease burden as the nation undergoes both an epidemiological and demographic transition [4]. Diverse epidemiological studies have identified risk factors for NCDs including unhealthy food consumption, use of tobacco, physical inactivity, and alcohol consumption. These risk factors contribute to four key metabolic changes: raised blood pressure (BP), high body weight, high blood glucose level (hyperglycemia), and high levels of fat in the blood (hyperlipidemia) leading to diabetes, hypertension, and overweight/obesity. The first nationally representative survey on NCDs of Bangladesh (Bangladesh Demographic and Health Survey [BDHS] 2011) showed that the prevalence rates of overweight/obesity, hypertension, and diabetes were 25.3%, 48.0%, and 11.0%, respectively [5].

It is important to know the changing pattern of NCDs over the years at the national level and to explore the distribution of NCDs among the different socioeconomic and demographic groups to identify common drivers of NCDs. Furthermore, to reduce the high prevalence and associated deaths of NCDs, evidence is needed on which demographic and socioeconomic factors are associated with changes in NCDs prevalence over time. The trend of NCDs has not been studied extensively in Bangladesh as only one study investigated the trend of NCDs, although it overlooked the variation in risk across socioeconomic groups [6]. Several studies in Bangladesh have examined associations among diabetes, hypertension and obesity, and their determinants but very few studies have used multiple waves of data to explain the changes over time. Only one study has been conducted to estimate the trends in obesity using multiple waves of BDHS [7]. One of the reasons might be the lack of nationally representative surveys on the prevalence of NCDs (i.e., hypertension and diabetes) in Bangladesh except for the BDHS’ 2011. Almost 8 years after the 2011 wave, the latest Bangladesh Demography and Health Survey, conducted in 2017–2018, collected information on the prevalence of NCDs. This provides a unique opportunity to study the trends in the prevalence and risk of NCDs across different socioeconomic groups in Bangladesh.

To achieve Sustainable Development Goal 3, aimed at reducing premature NCD‐related deaths by one third by 2030 [8], government requires accurate, timely, and reliable information on NCDs. Such data will enable evidence‐based decision‐making and formulation of tailored policies for populations affected by NCDs. The objective of this study is to [1] examine changes in NCDs (overweight and obesity, hypertension and diabetes) in Bangladesh from 2011 to 2018, both nationally and by region, [2] estimate the changes in the prevalence of NCDs within various population subgroups, with a particular focus on different socioeconomic strata, and [3] identify demographic and socioeconomic factors that are associated with NCDs.

## METHODS

### Data sources, sample size, and sampling method

We used data from the BDHS’ 2011 [5] and 2017 (BDHS’ 2017) [9], which are nationally representative and cross‐sectional. The surveys were carried out to collect information related to sociodemographic, anthropometric, health, and nutrition, by the National Institute for Population Research and Training of the Ministry of Health and Family Welfare in Bangladesh, in a collaboration with the ICF International (USA), and Mitra and Associates. The households were sampled using a two‐stage stratified sampling procedure. In the first stage, the probability proportional procedure was followed for the primary sampling unit, followed by a systematic selection procedure for the inclusion of the households in the second stage [5, 9].

A total of 18,000 households in 2011 and 20,250 households in 2017 were selected for the interviews with a response rate of 98% in both surveys. From these households, one third (6000) in 2011 and one fourth (5063) in 2017 were selected for the collection of biomarkers, that is, height, weight, BP, and blood glucose. Trained health technicians measured the fasting blood glucose (FBG) and BP following the guidelines approved by the Institutional Review Boards of Bangladesh. BP was measured three times at 10 min intervals using the LifeSource UA‐767 Plus BP monitor. The average of the second and third BP measurements was reported in the survey. To measure blood glucose using the HemoCue Glucose 201 RT system, participants were asked to fast at night for at least 8 h, and capillary blood was collected from the middle or ring finger in the early hours of the next day. Blood glucose was measured from the third drop of capillary blood after wiping away the first two drops. Weight was measured using a lightweight electronic SECA 878 scale that had a digital screen and ShorrBoard measuring board was used to measure height. More details of the sampling procedure and data collection information are described elsewhere [5, 9]. BDHS has been consistently following the same methodology for sampling design, sample size determination, data collection, and variable generation indicating that the variables are comparable between waves.

### Study population

**FIGURE 1:**
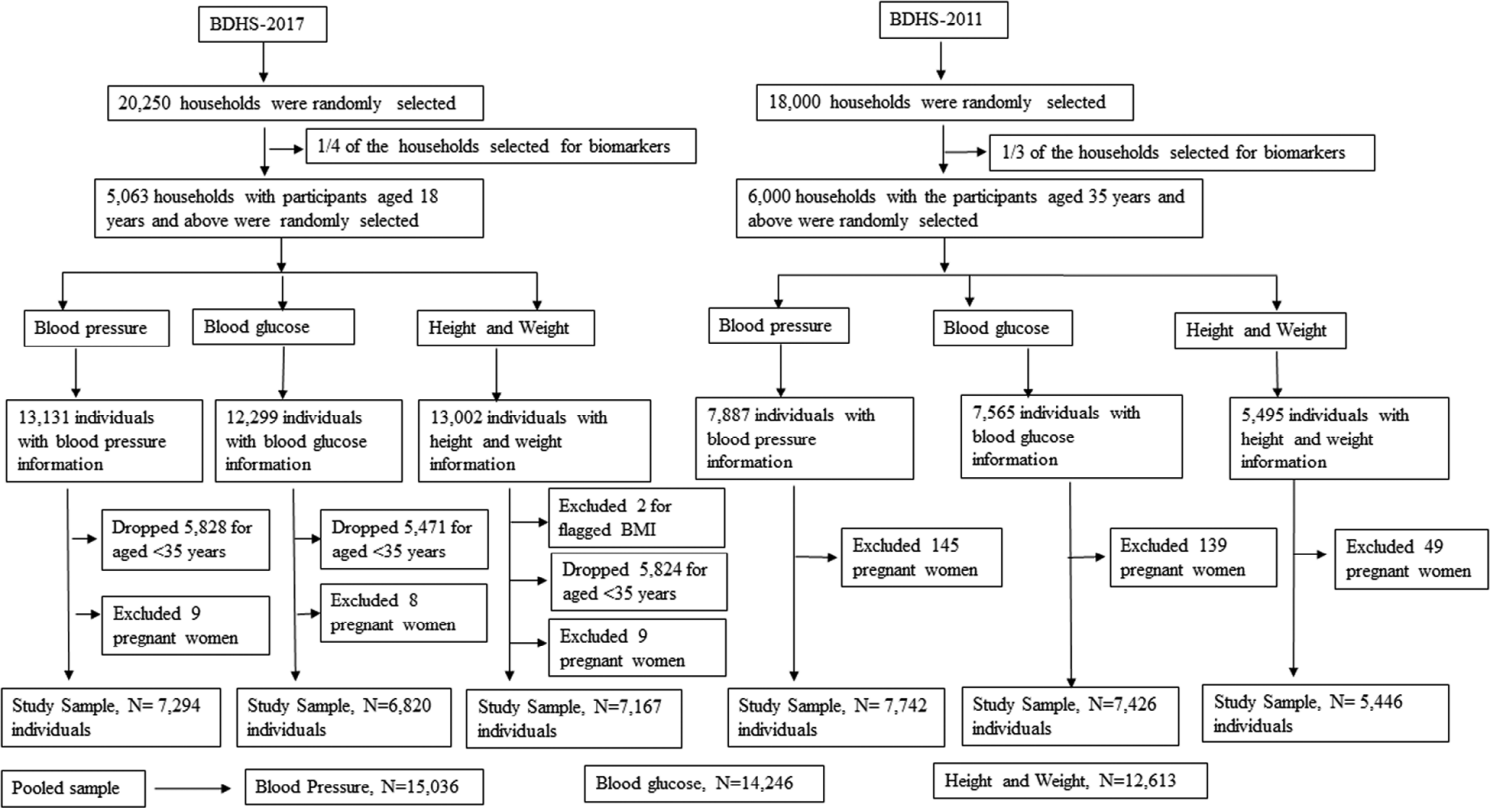
Flow diagram for the subject selection from Bangladesh Demographic and Health Survey (BDHS) 2011 and BDHS 2017.

In the BDHS’ 2011, information on biomarkers was collected from participants 35 years and above. However, in the BDHS’ 2017 survey, the eligibility for the biomarkers was 18 years old and above. For a better comparison between the two survey periods and to observe the trend, we only included participants aged 35 years and above from BDHS’ 2017. After the exclusion of refused, nonparticipant, and pregnant women, 7294 individuals with BP information, 6820 individuals with FBG information, and 7167 individuals with height and weight information are included from BDHS’ 2017. The corresponding sample size for BDHS’ 2011 is 7742 individuals with BP information, 7426 individuals with FBG information, and 5446 individuals with height and weight information. The sample selection procedure for the biomarkers is described in Figure 1.

### Statistical analysis

#### Dependent variable

Hypertension, diabetes, and overweight and obesity (overweight hereafter) are the main outcome variables of this study, which are derived from information on BP, FDG height, and weight. Definitions of dependent variables are presented in Table 1.

**TABLE 1:** Definitions of dependent variables.

Dependent variables	Definition	Reference
Hypertension	If participants were with ≥140 mm Hg or above‐average systolic blood pressure (SBP), or with ≥90 mm Hg or above‐average diastolic blood pressure (DBP) or taking any hypertensive medication in the survey time	WHO [40]
Diabetic	If participants either had FBG levels ≥7 mmol/L or reported having prescribed medicine for maintaining the blood glucose	WHO [41]
Overweight (and obesity)	If participants had BMI ≥25.00 kg/m^2^	WHO [42]

Abbreviation: FBG, fasting blood glucose.

#### Independent variable

The independent variables are gender, age, education, occupation, wealth index, and place and region of residence. Following previous work [10], participants up to 55 years of age were defined as younger, otherwise older. We defined participants’ education status into three categories: no education/preschool, primary (5+ years), and secondary/higher (10+ years). Occupation was divided into manual and nonmanual where sedentary workers (employed, businessman, landowner, drivers, students, unemployed, and retired) were considered nonmanual workers, whereas agricultural workers, daily labor, and working in poultry and other farms were considered manual workers. BDHS calculated the wealth index following the standard DHS wealth index method with the use of principal component analysis [9]. The wealth index was classified into quintiles: poorest, poorer, middle, richer, and richest. The place of residence was divided into urban and rural areas, whereas the region of residence followed Bangladesh's seven administrative divisions as of 2011.

We present the descriptive statistics as the number and percentage of the prevalence of hypertension, diabetes, and overweight. Although the prevalence of NCDs is not rare (>10%), estimating the prevalence ratio of NCDs while controlling for socioeconomic variables using the logistic model provides a poor estimate [11, 12]. The recommendations are to use Log binominal models or modified Poisson models [11, 12]. We chose the modified Poisson model as it produces a less biased estimate of the prevalence ratio compared to the log‐binominal model if the disease has a higher prevalence rate and moderate sample size. Additionally, the model provides unbiased estimates under model misspecification. We used a robust variance procedure to estimate standard errors [13, 14]. We adjusted for age in the regression analyses. Moreover, we adjusted also for the region of residence in the estimation of the prevalence rate, and region and place (rural/urban) of residence in the estimation of the national prevalence.

We performed three types of analyses: between survey years, within survey years, and a pooled analysis. In the between survey years analyses, we estimated age‐adjusted prevalence rate and the magnitude of NCDs using modified Poisson regression. For example, whether the prevalence rate of NCDs increased from 2011 to 2017 as well as the size of the increment. In the within survey years analyses, we estimated the prevalence ratio of NCDs for 2011 and 2017 separately for each independent variable while controlling for other factors using the modified Poisson regression model. For example, we estimated whether men have a higher prevalence of NCDs than women in 2011 and in 2017, separately. In the pooled analysis, we combined the two waves and used the 2011 wave as the reference.

We accounted for the complex survey design by applying the survey weights provided by the DHS for Bangladesh, and STATA 16 was used for the data management and analysis.

## RESULTS

Sociodemographic characteristics of the study population are described in Table 2. The percentage of people with hypertension, diabetes, and overweight has increased from 2011 to 2017, an increase that applies for all socioeconomic groups for overweight and hypertension.

**TABLE 2:** The sociodemographic characteristics of the study population in Bangladesh from 2011 to 2017.

	Overweight (and obesity) *N*		Hypertension		Diabetes	
	**2011 (*n* = 5446)**	**2017 (*n* = 7167)**	**2011 (*n* = 7742)**	**2017 (*n* = 7294)**	**2011 (*n* = 7426)**	**2017 (*n* = 6820)**
	***n* (%)**	***n* (%)**	***n* (%)**	***n* (%)**	***n* (%)**	***n* (%)**
**Total**	651 (12.0)	1852 (25.8)	2048 (26.4)	2947 (40.4)	863 (11.6)	922 (13.5)
**Sex**						
Male	411 (10.8)	671 (19.1)	793 (20.3)	1237 (34.8)	425 (11.4)	421 (12.7)
Female	240 (14.7)	1181 (32.3)	1255 (32.7)	1710 (45.7)	438 (11.9)	501 (14.3)
**Age (years)**						
Younger (<56 years)	411 (13.3)	1435 (30.0)	1111 (21.2)	1624 (33.8)	519 (10.3)	565 (12.6)
Older (≥56 years)	240 (10.2)	417 (17.5)	937 (37.5)	1323 (53.1)	344 (14.3)	357 (15.3)
**Education**						
No education, preschool	143 (5.8)	421 (15.2)	934 (26.6)	1142 (40.1)	287 (8.6)	275 (10.3)
Primary	162 (11.2)	560 (24.3)	500 (23.43)	896 (38.5)	236 (11.5)	282 (13.0)
Secondary and higher	346 (22.5)	871 (41.7)	614 (29.3)	909 (43.0)	340 (16.8)	364 (18.4)
**Occupation**						
Manual	84 (4.5)	539 (17.9)	262 (13.9)	966 (31.8)	121 (6.7)	237 (8.3)
Not manual	567 (15.8)	1308 (31.8)	1786 (30.51)	1974 (46.6)	742 (13.2)	682 (17.4)
**Wealth Index**						
Poorest	23 (2.3)	158 (11.0)	257 (18.7)	497 (34.1)	96 (7.3)	98 (7.1)
Poorer	37 (3.7)	217 (15.7)	307 (21.9)	493 (34.8)	100 (7.5)	100 (7.5)
Middle	71 (6.7)	282 (20.0)	336 (22.3)	565 (39.5)	109 (7.6)	145 (10.7)
Richer	147 (13.0)	394 (29.6)	451 (28.0)	576 (42.5)	170 (11.0)	196 (15.3)
Richest	373 (29.0)	801 (49.7)	697 (37.7)	816 (50.0)	388 (21.8)	383 (25.9)
**Place of residence**						
Rural	280 (7.6)	970 (20.7)	1226 (23.6)	1852 (38.7)	470 (9.4)	514 (11.4)
Urban	371 (21.1)	882 (35.6)	822 (32.3)	1095 (43.6)	393 (16.1)	408 (17.7)
**BMI status** (kg/m^2^)						
BMI < 25.00 kg/m^2^			1144 (23.9)	1843 (34.6)	464 (10.1)	535 (10.7)
BMI ≥ 25.00 kg/m^2^			302 (46.5)	1028 (55.5)	158 (25.6)	361 (21.1)
**Diabetes**						
Normal			1628 (24.9)	2266 (38.5)		
Diabetic			336 (39.0)	511 (55.5)		
**Region**						
Barisal	57 (8.7)	209 (26.6)	232 (25.1)	374 (46.5)	112 (13.1)	98 (13.1)
Chittagong	116 (14.1)	304 (33.1)	266 (23.0)	405 (43.3)	157 (14.4)	149 (17.2)
Dhaka	99 (10.7)	451 (25.9)	364 (27.4)	634 (35.9)	152 (11.7)	254 (15.8)
Khulna	121 (14.2)	314 (29.5)	368 (30.2)	450 (41.4)	105 (8.9)	131 (12.7)
Rajshahi	107 (14.6)	216 (23.6)	282 (25.7)	368 (39.4)	120 (11.5)	109 (12.3)
Rangpur	67 (8.6)	194 (20.6)	323 (30.2)	400 (42.0)	92 (8.8)	79 (8.6)
Sylhet	84 (12.4)	164 (20.5)	213 (22.4)	316 (38.8)	125 (13.8)	102 (13.6)

**FIGURE 2:**
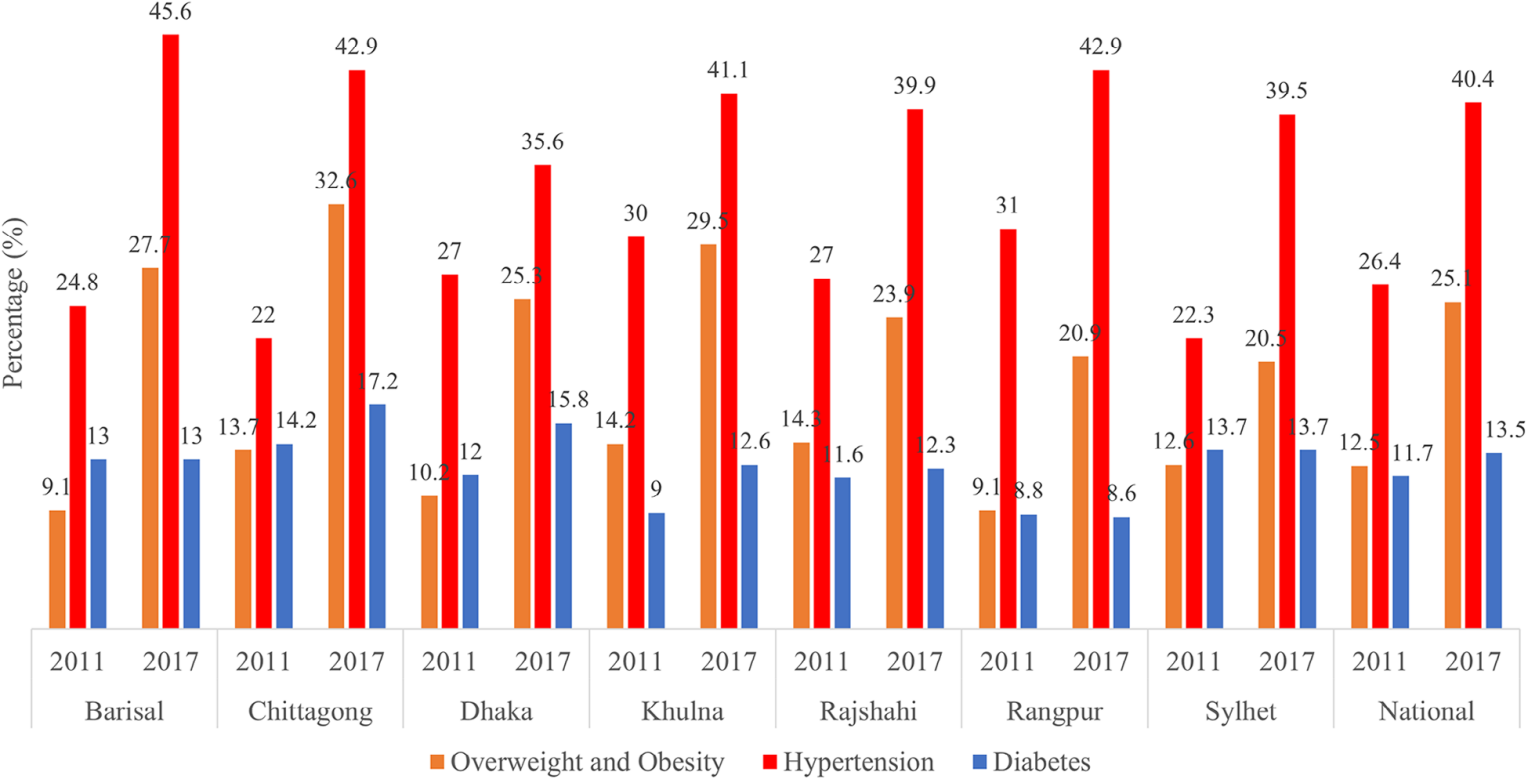
Age‐adjusted prevalence of overweight and obesity, hypertension, and diabetes by divisions of Bangladesh from 2011 to 2017.

Figure 2 presents the regional and the national age‐adjusted prevalence rates of NCDs. The national prevalence rates of overweight and hypertension increased by 12.6% and 14.0% between 2011 and 2017, whereas for diabetes the increase was 1.8%. The prevalence of overweight and hypertension has increased in all regions, whereas the prevalence of diabetes only increased slightly in Chittagong, Dhaka, Khulna, and Rajshahi regions.

In Table 3, we present the results for the between years analyses. We found a significant increase in the prevalence rate of overweight in all socioeconomic groups. The highest increase in relative terms was in the poorest group where the age‐adjusted prevalence rate increased from 2.4% in 2011 to 11.1% in 2017, or 4.4 times. Manual workers and the second poorest wealth group had the second highest increase (3.8 times). In terms of hypertension, a significant increase across all the socioeconomic groups was found. However, the highest relative increase at 2.4 times was noted for manual workers. In contrast, diabetes only increased for some socioeconomic groups with the highest relative increase for middle‐ and richer wealth groups at 1.4 times. Although not statistically significant, the prevalence decreased in the poorest group and among people who are not overweight (Table 3).

**TABLE 3:** Age‐adjusted prevalence rate of overweight and obesity, hypertension, and diabetes by characteristics in Bangladesh from 2011 to 2017 (between survey year analyses).

	Overweight and obesity (95% CI)				Hypertension (95% CI)				Diabetes (95% CI)			
	2011 (*n* = 5446)	2017 (*n* = 7167)	Prevalence ratio (PR)		2011 (*n* = 7742)	2017 (*n* = 7294)	Prevalence ratio (PR)		2011 (*n* = 7426)	2017 (*n* = 6820)	Prevalence ratio (PR)	
	Prevalence rate	Prevalence rate	PR	*p*‐Value	Prevalence rate	Prevalence rate	PR	*p*‐Value	Prevalence rate	Prevalence rate	PR	*p*‐Value
Sex												
Male	10.1 (9–11)	19.2 (18–21)	1.8 (1.6–2.0)	<0.001	20.1 (20–21)	34.7 (33–36)	1.7 (1.6–1.9)	<0.001	11.3 (10–12)	12.7 (12–14)	1.2 (1.0–1.3)	0.076
Female	17.7 (15–20)	32.1 (31–34)	1.6 (1.4–1.9)	<0.001	33 (32–35)	45.9 (44–48)	1.4 (1.3–1.5)	<0.001	11.9 (11–13)	14.3 (13–16)	1.2 (1.1–1.4)	0.003
Education												
No education, preschool	5.8 (5–7)	16.1 (15–18)	2.5 (2.1–3.0)	<0.001	24.6 (23–26)	36.6 (35–38)	1.5 (1.4–1.6)	<0.001	8.2 (7–9)	9.7 (9–11)	1.2 (1.0–1.4)	0.030
Primary	11.2 (10–13)	23.7 (22–25)	2.1 (1.8–2.5)	<0.001	24.5 (23–26)	40.2 (38–42)	1.7 (1.5–1.8)	<0.001	11.8 (10–13)	13.4 (12–15)	1.1 (1.0–1.3)	0.133
Secondary and higher	22.3 (20–24)	39.8 (38–42)	1.8 (1.6–2.0)	<0.001	32.3 (30–34)	46.8 (45–49)	1.5 (1.4–1.6)	<0.001	17.8 (16–20)	19.5 (18–21)	1.1 (1.0–1.3)	0.173
Occupation												
Manual	4.1 (3–5)	17.1 (16–18)	3.8 (3.0–4.7)	<0.001	14.4 (13–16)	33.4 (32–35)	2.4 (2.1–2.7)	<0.001	6.8 (6–8)	8.4 (7–9)	1.2 (1.0–1.5)	0.043
Not manual	16.6 (15–18)	32.8 (31–34)	1.8 (1.7–2.0)	<0.001	30.1 (29–31)	45.1 (44–47)	1.5 (1.4–1.6)	<0.001	13.1 (12–14)	17.2 (16–18)	1.3 (1.2–1.4)	<0.001
Wealth index												
Poorest	2.4 (1–3)	11.1 (10–13)	4.4 (2.9–6.7)	<0.001	18.4 (16–20)	33.8 (32–36)	1.8 (1.6–2.1)	<0.001	7.2 (6–9)	7.1 (6–8)	0.9 (0.7–1.3)	0.891
Poorer	3.7 (3–5)	15.7 (14–18)	3.8 (2.7–5.3)	<0.001	22 (20–24)	34.7 (32–37)	1.6 (1.4–1.8)	<0.001	7.5 (6–9)	7.5 (6–9)	1.0 (0.8–1.3)	0.986
Middle	6.7 (5–8)	20.1 (18–22)	2.7 (2.1–3.5)	<0.001	22.1 (20–24)	39.3 (37–42)	1.8 (1.6–2.0)	<0.001	7.5 (6–9)	10.7 (9–12)	1.4 (1.1–1.8)	0.004
Richer	13.1 (11–15)	29.6 (27–32)	2.1 (1.8–2.5)	<0.001	27.6 (26–30)	42.5 (40–45)	1.5 (1.4–1.7)	<0.001	10.9 (9–12)	15.3 (13–17)	1.4 (1.2–1.7)	0.001
Richest	28.7 (26–31)	49.4 (47–52)	1.7 (1.5–1.8)	<0.001	38.8 (37–41)	50.6 (48–53)	1.3 (1.2–1.4)	<0.001	22.1 (20–24)	26 (24–28)	1.2 (1.0–1.3)	0.010
Place of residence												
Rural	7.6 (7–9)	20.9 (20–22)	2.5 (2.2–2.8)	<0.001	23.3 (22–24)	38.3 (37–40)	1.6 (1.5–1.7)	<0.001	9.4 (9–10)	11.3 (10–12)	1.2 (1.1–1.4)	0.002
Urban	20.8 (19–23)	34.9 (33–37)	1.6 (1.5–1.8)	<0.001	33.1 (31–35)	44.6 (43–47)	1.4 (1.3–1.5)	<0.001	16.3 (15–18)	17.9 (16–20)	1.1 (0.9–1.2)	0.139
BMI status (kg/m^2^)												
BMI < 25.00 kg/m^2^					23.7 (23–25)	33.8 (33–35)	1.6 (1.5–1.7)	<0.001	10.1 (9–11)	10.6 (10–11)	1.1 (0.9–1.2)	0.147
BMI ≥ 25.00 kg/m^2^					50.2 (46–54)	60.2 (58–63)	1.3 (1.2–1.4)	<0.001	26.4 (23–30)	22.1 (20–24)	0.9 (0.8–1.1)	0.284
Diabetes												
Normal					25 (24–26)	38.6 (37–40)	1.5 (1.5–1.6)	<0.001				
Diabetic					37.7 (35–41)	54 (51–57)	1.4 (1.3–1.6)	<0.001				

In Table 4, we present the within years analyses. We note that the strength of the association between overweight and socioeconomic status has fallen between the waves. For example, in 2011, people in the richest household had 5.6 times higher prevalence of overweight compared to the poorest, whereas, in 2017, it has fallen to three times. The same trend is noted for hypertension, where, for example, nonmanual workers had 1.5 times higher prevalence of having hypertension compared to manual workers in 2011, compared to 1.2 times in 2017. For diabetes, we see mixed results. The strength of the association has fallen for younger (<56 years) and for persons with BMI < 25, whereas it has increased with regard to wealth index. In 2011, the richest group had 1.9 times higher prevalence of diabetes compared to the poorest, compared to 2.6 times in 2017.

**TABLE 4:** Socioeconomic factors associated with overweight and obesity, hypertension, and diabetes in Bangladesh from 2011 to 2017 (within).

	Overweight and obesity (95% CI)				Hypertension (95% CI)				Diabetes (95% CI)			
	2011 (*n* = 5446)		2017 (*n* = 7167)		2011 (*n* = 7742)		2017 (*n* = 7294)		2011 (*n* = 7426)		2017 (*n* = 6820)	
Variables	Prevalence ratio (PR)	*p*‐Value	Prevalence ratio (PR)	*p*‐Value	Prevalence ratio (PR)	*p*‐Value	Prevalence ratio (PR)	*p*‐Value	Prevalence ratio (PR)	*p*‐Value	Prevalence ratio (PR)	*p*‐Value
Sex												
Male	1		1		1		1		1		1	
Female	1.8 (1.7–2.2)	<0.001	1.8 (1.9–2.0)	<0.001	1.6 (1.4–1.7)	<0.001	1.2 (1.2–1.3)	<0.001	1.0 (0.9–1.2)	0.985	1.1 (0.9–1.2)	0.457
Education												
No education, preschool	1		1		1		1		1		1	
Primary	1.7 (1.3–2.1)	<0.001	1.4 (1.2–1.5)	<0.001	1.0 (0.9–1.1)	0.504	1.0 (0.9–1.1)	0.610	1.3 (1.0–1.5)	0.033	1.2 (1.0–1.4)	0.059
Secondary and higher	2.3 (1.9–2.9)	<0.001	1.9 (1.6–2.0)	<0.001	1.1 (1.0–1.2)	0.254	1.0 (0.9–1.1)	0.641	1.4 (1.1–1.7)	0.004	1.1 (0.9–1.3)	0.274
Occupation												
Manual	1		1		1		1		1		1	
Not manual	1.7 (1.4–2.2)	<0.001	1.2 (1.1–1.3)	<0.001	1.5 (1.3–1.7)	<0.001	1.2 (1.1–1.3)	<0.001	1.4 (1.1–1.7)	0.002	1.4 (1.2–1.7)	<0.001
Wealth Index												
Poorest	1		1		1		1		1		1	
Poorer	1.4 (0.8–2.3)	0.210	1.3 (1.1–1.6)	0.004	1.1 (0.9–1.3)	0.330	1.0 (0.9–1.1)	0.977	0.9 (0.7–1.3)	0.709	1.0 (0.8–1.4)	0.802
Middle	2.2 (1.4–3.4)	0.001	1.5 (1.3–1.8)	<0.001	1.2 (1.0–1.4)	0.053	1.1 (1.0–1.2)	0.019	0.8 (0.6–1.1)	0.103	1.4 (1.1–1.8)	0.019
Richer	3.4 (2.2–5.2)	<0.001	2.0 (1.7–2.4)	<0.001	1.4 (1.2–1.6)	<0.001	1.1 (1.0–1.3)	0.008	1.0 (0.8–1.4)	0.785	1.8 (1.4–2.2)	<0.001
Richest	5.6 (3.6–8.7)	<0.001	3.0 (2.5–3.5)	<0.001	1.6 (1.4–1.9)	<0.001	1.2 (1.1–1.3)	0.005	1.9 (1.4–2.5)	<0.001	2.6 (2.0–3.3)	<0.001
Place of residence												
Rural	1		1		1		1		1		1	
Urban	1.3 (1.1–1.5)	<0.001	1.1 (1.0–1.2)	0.230	1.1 (1.0–1.2)	0.232	1.00 (0.9–1.1)	0.990	1.0 (0.9–1.2)	0.747	1.0 (0.9–1.1)	0.650
BMI status (kg/m^2^)												
BMI < 25.00 kg/m^2^					1		1		1		1	
BMI ≥ 25.00 kg/m^2^					1.5 (1.3–1.7)	<0.001	1.5 (1.4–1.6)	<0.001	1.7 (1.5–2.0)	<0.001	1.4 (1.3–1.6)	<0.001
Diabetes												
Normal					1		1					
Diabetic					1.2 (1.1–1.3)	0.001	1.2 (1.2–1.3)	<0.001				
Age												
Younger (<56 years)	1		1		1		1		1		1	
Older (≥56 years)	0.8 (0.7–0.9)	0.001	0.7 (0.6–0.8)	<0.001	1.7 (1.5–1.9)	<0.001	1.6 (1.5–1.7)	<0.001	1.6 (1.4–1.8)	<0.001	1.3 (1.1–1.4)	0.001
Region												
Rajshahi	1		1		1		1		1		1	
Barisal	0.7 (0.5–0.9)	0.004	1.1 (0.9–1.3)	0.249	0.9 (0.7–1.0)	0.092	1.1 (1.0–1.2)	0.130	1.2 (0.9–1.5)	0.277	1.0 (0.8–1.3)	0.770
Chittagong	0.9 (0.7–1.2)	0.426	1.1 (1.0–1.3)	0.160	0.8 (0.7–0.9)	0.001	1.0 (0.9–1.1)	0.621	1.1 (0.9–1.5)	0.302	1.1 (0.9–1.4)	0.500
Dhaka	0.7 (0.5–0.8)	<0.001	1.0 (0.8–1.1)	0.551	0.9 (0.8–1.1)	0.360	0.9 (0.8–1.0)	0.004	0.9 (0.7–1.2)	0.524	1.2 (0.9–1.4)	0.166
Khulna	1.0 (0.8–1.2)	0.746	1.1 (1.0–1.3)	0.214	1.1 (0.9–1.2)	0.354	1.0 (0.9–1.1)	0.598	0.7 (0.5–0.9)	0.005	0.9 (0.7–1.1)	0.289
Rangpur	0.8 (0.6–1.1)	0.121	1.0 (0.8–1.1)	0.643	1.2 (1.0–1.4)	0.067	1.1 (1.0–1.2)	0.071	0.8 (0.6–1.1)	0.208	0.9 (0.6–1.0)	0.085
Sylhet	0.8 (0.6–1.0)	0.033	0.8 (0.7–0.9)	0.006	0.8 (0.6–0.9)	0.004	1.0 (0.9–1.1)	0.612	1.2 (0.9–1.5)	0.285	1.0 (0.8–1.3)	0.842

In the pooled analyses (Table 5), we found that the NCDs has increased over the two waves, overweight by 1.8 times, hypertension by 1.5 times, and diabetes by 1.1 times. People from the richest households have a higher risk of NCDs compared to the poorest, 3.3 times for overweight, 2.3 times for diabetes, and 1.3 times for hypertension. Moreover, individuals with overweight are 1.5 times more likely to have hypertension and diabetes compared to the normal weight.

**TABLE 5:** Socioeconomic factors associated with overweight and obesity, hypertension, and diabetes in Bangladesh (pooled analysis).

	Overweight (95% CI)		Hypertension (95% CI)		Diabetes (95% CI)	
Variables	Prevalence ratio (PR)	*p*‐Value	Prevalence ratio (PR)	*p*‐Value	Prevalence ratio (PR)	*p*‐Value
Sex						
Male	1		1		1	
Female	1.8 (1.7–2.0)	<0.001	1.3 (1.2–1.4)	<0.001	1.0 (0.9–1.2)	0.438
Education						
No education, preschool	1		1		1	
Primary	1.5 (1.3–1.6)	<0.001	1.0 (0.9–1.1)	0.525	1.2 (1.1–1.4)	0.005
Secondary and higher	1.9 (1.8–2.2)	<0.001	1.0 (1.0–1.1)	0.281	1.2 (1.1–1.4)	0.005
Occupation						
Manual	1		1		1	
Not manual	1.3 (1.2–1.4)	<0.001	1.3 (1.2–1.3)	<0.001	1.4 (1.2–1.9)	<0.001
Wealth Index						
Poorest	1		1		1	
Poorer	1.3 (1.1–1.6)	0.003	1.0 (0.9–1.1)	0.619	1.0 (0.8–1.2)	0.910
Middle	1.6 (1.3–1.8)	<0.001	1.1 (1.0–1.2)	0.005	1.1 (0.9–1.3)	0.336
Richer	2.2 (1.9–2.6)	<0.001	1.2 (1.1–1.3)	<0.001	1.4 (1.2–1.7)	<0.001
Richest	3.3 (2.8–3.9)	<0.001	1.3 (1.2–1.4)	<0.001	2.3 (1.9–2.7)	<0.001
Place of residence						
Rural	1		1		1	
Urban	1.1 (1.0–1.2)	0.002	1.0 (1.0–1.1)	0.411	1.0 (0.9–1.1)	0.934
BMI status (kg/m^2^)						
BMI < 25.00 kg/m^2^			1		1	
BMI ≥ 25.00 kg/m^2^			1.5 (1.4–1.6)	<0.001	1.5 (1.4–1.7)	<0.001
Diabetes						
Normal			1			
Diabetic			1.2 (1.1–1.3)	<0.001		
Age (years)						
Younger (<56 years)	1		1		1	
Older (≥56 years)	0.7 (0.6–0.8)	<0.001	1.7 (1.6–1.8)	<0.001	1.4 (1.3–1.5)	<0.001
Region						
Rajshahi	1		1		1	
Barisal	0.9 (0.8–1.1)	0.379	1.0 (0.9–1.1)	0.997	1.1 (0.9–1.3)	0.465
Chittagong	1.0 (0.9–1.2)	0.729	0.9 (0.8–1.0)	0.007	1.1 (0.9–1.3)	0.280
Dhaka	0.9 (0.8–1.0)	0.012	0.9 (0.8–1.0)	0.001	1.1 (0.9–1.3)	0.417
Khulna	1.0 (0.9–1.2)	0.561	1.0 (0.9–1.1)	0.961	0.9 (0.7–0.9)	0.009
Rangpur	0.9 (0.8–1.0)	0.115	1.1 (1.0–1.2)	0.016	0.8 (0.7–1.0)	0.026
Sylhet	0.8 (0.7–0.9)	0.001	0.9 (0.8–1.0)	0.021	1.1 (0.9–1.3)	0.551
Survey year						
2011	1		1		1	
2017	1.8 (1.7–2.0)	<0.001	1.5 (1.4–1.6)	<0.001	1.1 (1.0–1.2)	0.53

## DISCUSSION

In this study, we estimated the prevalence of NCDs (e.g., overweight and obesity, hypertension, and diabetes) in Bangladesh and changes in prevalence between 2011 and 2017, as well as investigated the association of socioeconomic factors with the prevalence of NCDs using nationally representative surveys.

Our findings indicate a rising trend in overweight prevalence, with an increase of approximately 12.6% from 2011 to 2017. Some prominent risk factors for being overweight are unhealthy diet, physical inactivity, and economic growth [15]. Bangladesh has been experiencing sustained economic growth, with the real GDP growth rate increasing from 5.2% in 2011 to 7.0% in 2017 [16]. Researchers have shown that in Bangladesh, modern diet is associated with higher income per capita [17], and with the rapid economic growth, fast food and processed food are now readily available. Intake of fruits and vegetables is also low, with rates up to 90% consuming inadequate amount of fruits and vegetables [18]. It is likely that a calorie‐dense diet with a low intake of fruits and vegetables is a part of the explanation for the increasing trend of overweight and obesity in Bangladesh.

Another prominent factor for overweight is physical inactivity [19]. The level of physical inactivity in Bangladesh is unclear, a scoping review indicated a range between 5% and 83% [20], whereas a corresponding figure for the elderly is 40% [21]. However, a modern lifestyle is associated with increased sedentary behavior and is expected to contribute to the rising prevalence of overweight in Bangladesh.

Our finding that the highest increase in obesity is observed among the poorest and least educated segments of the population aligns with previous findings [22], also from India and other LMICs [23, 24]. There are many potential explanations for this trend, for example, the inverse relationship between food cost and energy density. Energy‐dense foods composed of refined grains, sugar, and fats often represent the lowest cost option for the poor as more prudent diets based on lean meats, fish, fresh fruits, and vegetables are more expensive [15, 25]. The low‐cost high‐energy consumption among poor people is expected to contribute to weight gain by increasing calorie intake [26].

Another possible explanation is the transformation of manual labor transportation into motorized transportation. Rickshaw pulling was one of the main occupations for poor people in Bangladesh [27]. Over the last decade, manual rickshaws have gradually been replaced by battery‐operated rickshaws, with approximately 200,000 of these vehicles currently in service in Dhaka city [27]. This increased motorization in the Bangladeshi society is expected to contribute to reduced physical activity among adults.

Our findings of an increase in the prevalence of hypertension are consistent with a systematic review and meta‐analysis of studies from Bangladesh [28]. It is alarming that persons in manual labor as well as the poorest, especially women, have the highest increase in the prevalence ratio of hypertension between 2011 and 2017. Some of the established risk factors for hypertension are obesity, unhealthy diet (including low fruits and vegetables and high salt intake), stress, and air pollution [29]. As discussed above, manual workers and the poorest people, especially women, have had the highest increase in the prevalence of overweight and obesity between 2011 and 2017. This higher rate of obesity is expected to contribute to the increase in the prevalence of hypertension, and relatively more so among those in manual labor, the poorest, and women compared to those in nonmanual, the richer, and men.

Another possible explanation might be the air quality of Bangladesh as air pollution has been established as a major risk factor of high BP [30]. Overall, the air quality of Bangladesh is unhealthy and 15.4 times more polluted compared to the WHO annual air quality value [31], and the situation, especially in the capital, has worsened during the last two decades [32]. Poor people and manual labor are more exposed to air pollution for their economic and daily activities compared to rich people, and air pollution could thus potentially contribute to the noted increase in hypertension.

We have found an increasing trend of diabetes in Bangladeshi adults, which is in line with a previous study from Bangladesh [33]. Both obesity and hypertension are common risk factors for diabetes, and given the observed increase in obesity and hypertension, it is not surprising that the prevalence of diabetes has increased in Bangladesh. It is interesting to note that the prevalence has increased in rural areas of Bangladesh, whereas no statistically significant increase is noted for urban areas. The reasons might be that the intake of locally produced vegetables and cereals has decreased in rural areas over the years, and a diet shift can be observed toward high‐fat, high‐energy‐dense modern diets. Together with the advancement of modernization and an increased sedentary lifestyle, as discussed above, this is likely to contribute to the increased rate of diabetes in rural areas. Studies from other countries with the similar socioeconomic and cultural backgrounds as Bangladesh have shown an increasing incidence of diabetes in the rural populations [34, 35].

We also find that the trend of increasing diabetes is higher among people from the higher socioeconomic class. This is also not uncommon as rich people generally have low levels of physical activity and have higher comorbidities (hypertension and obesity) [36] than poor people. All these are the risk factors for diabetes, and the similar trend has been found in LMICs as shown in a meta‐analysis [22].

We also find divisional variance in the prevalence of NCDs across Bangladesh's eight administrative divisions. There are some distinctive features in the divisions as the geography of Bangladesh is diverse with plain land in the middle (Dhaka division), small hills in the southeast (Chattogram division), low hills in the northeast (Sylhet division), highlands in the north and northwest (Rajshahi division), and numerous rivers with associated flooding in the south (Barisal and Khulna divisions) [37]. Besides these geographical variations, there are socioeconomic variations with the richer Dhaka and Chattogram divisions. Regional variations in terms of NCDs are thus understandable given the context‐specific aspect of risk factors.

This study has potential to inform the policymakers about the current situation and trend of overweight, hypertension, and diabetes in Bangladesh. People living in rural areas and in low socioeconomic groups are rapidly becoming obese and hypertensive. The government of Bangladesh prioritizes tackling the NCDs with a key action plan in its recent Health, Nutrition, and Population Sector Intervention Program (2016–2021) and highlights a multisectoral approach to promoting healthy lifestyles and environments [38]. However, inadequate planning, implementation, and monitoring of the policy activities have been identified as weaknesses of the policy [39]. Therefore, special attention is required for rural areas and for the poorest people in order to combat this alarming trend in the future.

A strength of the current study is that it is the first study to investigate the prevalence ratio of overweight, hypertension, and diabetes both between and within two waves of a large nationally representative survey in Bangladesh. Data for BP, blood glucose, and anthropometric measurement (weight and height) were collected by the expert health technicians following the standard guidelines. It should be noted that we have used the WHO cut‐off for overweight and obesity classification instead of Asian cut‐off to align with other BDHS reports and international comparisons. A limitation of the study is the cross‐sectional nature of the data, and the resulting study of associations. In addition, some factors that may have affected the NCDs, such as blood lipid profile, waist and hip circumference, dietary behaviors, and physical activity information, are not available in the surveys.

## CONCLUSIONS

This study highlights a concerning upward trend in the prevalence of NCDs in Bangladesh. From 2011 to 2017, overweight, obesity, and hypertension have increased especially in rural areas, among poor and manual workers, although high socioeconomic groups still have the greater risk of NCDs. The growing burden of NCDs is exerting significant pressure on the healthcare system. To prevent further increase in NCDs in Bangladesh, a robust strategic policy and awareness program is imperative, with a particular emphasis on educating and raising awareness among the rural and underserved populations.

## AUTHOR CONTRIBUTIONS

Sanjib Saha and Masum Ali conceptualized the study and prepared the initial manuscript. Masum Ali analyzed the data. Md. Ruhul Amin and Johan Jarl reviewed the manuscript. All authors critically reviewed the final manuscript.

## CONFLICT OF INTEREST STATEMENT

The authors declared that there are no conflicts of interest exist.

## ETHICS STATEMENT

We used de‐identified secondary datasets from the Demographic Health Survey (DHS), and the datasets are freely available from the DHS for research. Thus, ethical approval is not applicable for this.

## Data Availability

The dataset used for this study is publicly for the researchers from the Demography and Health surveys (https://dhsprogram.com/Countries/Country‐Main.cfm?ctry_d=1&c=Bangladesh&Country=Bangladesh&cn=&r=4).
